# Utilizing Ion Mobility-Mass Spectrometry to Investigate the Unfolding Pathway of Cu/Zn Superoxide Dismutase

**DOI:** 10.3389/fchem.2021.614595

**Published:** 2021-02-09

**Authors:** Karen E. Butler, Yoshihiko Takinami, Adam Rainczuk, Erin S. Baker, Blaine R. Roberts

**Affiliations:** ^1^ Department of Chemistry, North Carolina State University, Raleigh, NC, United States; ^2^ Bruker Japan K.K., Yokohama City, Japan; ^3^ Bruker Pty Ltd., Preston, VIC, Australia; ^4^ Department of Biochemistry, Emory University School of Medicine, Atlanta, GA, United States; ^5^ Department of Neuroscience, Emory University School of Medicine, Atlanta, GA, United States

**Keywords:** ion mobility spectrometry, native mass spectrometry, superoxide dismutase, SOD1, drift tube ion mobility spectrometry, trapped ion mobility spectrometry

## Abstract

Native mass spectrometry has emerged as a powerful tool for structural biology as it enables the evaluation of molecules as they occur in their physiological conditions. Ion mobility spectrometry-mass spectrometry (IMS-MS) has shown essential in these analyses as it allows the measurement of the shape of a molecule, denoted as its collision cross section (CCS), and mass. The structural information garnered from native IMS-MS provides insight into the tertiary and quaternary structure of proteins and can be used to validate NMR or crystallographic X-ray structures. Additionally, due to the rapid nature (millisecond measurements) and ability of IMS-MS to analyze heterogeneous solutions, it can be used to address structural questions not possible with traditional structural approaches. Herein, we applied multiple solution conditions to systematically denature bovine Cu/Zn-superoxide dismutase (SOD1) and assess its unfolding pathway from the holo-dimer to the holo-monomer, single-metal monomer, and apo-monomer. Additionally, we compared and noted 1–2% agreement between CCS values from both drift tube IMS and trapped IMS for the SOD1 holo-monomer and holo-dimer. The observed CCS values were in excellent agreement with computational CCS values predicted from the homo-dimer crystal structure, showcasing the ability to use both IMS-MS platforms to provide valuable structural information for molecular modeling of protein interactions and structural assessments.

## Introduction

The elucidation of protein structures is of great interest to biochemists and structural biologists, as their specific interactions and arrangements have an intimate association with their function. As such, investigations of protein structure can provide information about the roles of protein folding and complexation in biological processes and disease states. While the conformation of proteins in their native state has traditionally been investigated using biophysical techniques such as X-ray crystallography, cryo-electron microscopy (cryo-EM), and nuclear magnetic resonance (NMR), advancements in native mass spectrometry (MS) have led to its emergence as another important tool in native structural assessments. Native MS offers many advantages over the more traditional methods as it requires less sample compared to NMR or X-ray crystallography, is more tolerant of heterogeneous samples, and allows the analysis of protein structures that fail to take on the orderly arrangement necessary for crystallization. (Heck and van den Heuvel, 2004; Konijnenberg et al., 2013; Leney and Heck, 2017).

While native MS is an excellent tool for probing protein-ligand complexation or protein binding, MS alone lacks the ability to give definite measurements of the relative molecular size of those complexes. Thus, many native MS experiments are performed by coupling ion mobility spectrometry and MS (IMS-MS). IMS lends itself as a powerful complement to native MS analyses, as the simultaneous measurements allow for the separation of analytes based on size, charge, and mass in a millisecond time frame (Bohrer et al., 2008). A variety of IMS-MS platforms have been used to assess the conformation of biomolecules, and while the application of the electric field and gas flow may vary, the general principles of IMS are conserved (Wei et al., 2019). Overall, IMS is a gas-phase separation technique that measures the mobility of a gaseous ion through an inert buffer gas, typically nitrogen or helium, and under the influence of an electric field (Mason and Schamp, 1958). The ion mobility measurements can then be used to determine the ion-neutral collision cross-section (CCS), a molecular descriptor that gives information about the size of a molecule (Mason and Schamp, 1958; Revercomb and Mason, 1975).

The exploration of protein structure using IMS-MS has been used to thoroughly investigate the conformational landscape of many proteins, including ubiquitin (Wyttenbach and Bowers, 2011), cytochrome c (May et al., 2018), and amyloid protein (Wyttenbach et al., 2014). Comparisons between crystallography and IMS-MS studies have shown correlation between the CCS values predicted from crystal structures and those observed in IMS-MS experiments for many proteins, demonstrating that tertiary protein structure is conserved during electrospray ionization in many cases (Jurneczko and Barran, 2011; Rolland and Prell, 2019). Furthermore, the use of IMS-MS yields structural information beyond what could be inferred from charge state distributions alone, as it allows for the analysis of multiple conformers within the same charge state (Smith et al., 2007). The ability to analyze both compact and extended forms of the same protein simultaneously has opened many doors for the study of native proteins and has made IMS-MS a suitable method for the evaluation of molecular dynamics in protein folding studies (Smith et al., 2007; Wyttenbach and Bowers, 2011; Lanucara et al., 2014; Wyttenbach et al., 2014).

In this study, we investigated acid-induced unfolding of bovine Cu/Zn-superoxide dismutase (SOD1). SOD1 is a ubiquitous antioxidant enzyme that serves to protect cells from oxidative damage through the dismutation of superoxide into hydrogen peroxide and oxygen (McCord and Fridovich, 1969). In its active form, it is observed to be a highly soluble 32 kDa dimeric metalloprotein comprised of two monomers each with a single disulfide bridge (Abernethy et al., 1974; Richardson et al., 1975). Each monomer binds one copper ion, which acts as a catalyst for the enzyme, and one zinc ion, which primarily promotes protein stability (Tainer et al., 1983; Nedd et al., 2014). As dysregulation of this protein is implicated in neurodegenerative conditions including Parkinson’s disease (Choi et al., 2005; Trist et al., 2017; Tokuda et al., 2019), Alzheimer’s disease (Choi et al., 2005), and amyotrophic lateral sclerosis (ALS) (Rakhit et al., 2004; Nordlund and Oliveberg, 2008; Tokuda et al., 2019), the structure has been heavily investigated. In the case of ALS, to date over 150 mutant forms of SOD have been identified as potential ALS-promoting mutations (http://alsod.iop.kcl.ac.uk) (Abel et al., 2012). In general, the mutations promote a gain in function and although it is not clear which mechanism is responsible for the disease phenotype, the mutations do promote protein misfolding, incomplete metalation, and aggregation. Thus, the native characterization of the folding behavior of this protein in differing degrees of metalation is crucial to understanding its nature *in vivo*. Due to both the presence of a disulfide bridge and metal cofactors, SOD1 is a highly stable metalloprotein. However, if the disulfide bridge or presence of the metal co-factors is compromised, the SOD1 monomers are more likely to dissociate and lose their metal cofactors (Roberts et al., 2007; Sahawneh et al., 2010). Several research groups have attempted to use IMS-MS to probe parts of this unfolding pathway to better characterize intermediates between the different protein states. One such study, conducted by Zhuang and coworkers, reported on how voltage systematically denatured bovine SOD1 from the holo-dimer, holo-monomer, and apo-monomer (Zhuang et al., 2014). The researchers observed three different dimeric and two monomeric conformers when analyzing the samples using IMS-MS/MS, leading them to propose that three separate unfolding and dissociation pathways were utilized by the enzyme (Zhuang et al., 2014). Additional work by McAlary et al. used collision induced unfolding (CIU) combined with IMS-MS to examine several mutations of human SOD1 compared with the wild-type SOD1 and found that six of the seven mutants unfolded via two unfolding events (McAlary et al., 2020). The seventh mutant, G37R, was observed to asymmetrically dissociate, and was ultimately determined to have higher energy requirements than the other variants for unfolding (McAlary et al., 2020).

In this manuscript, we utilized both a commercially available drift tube IMS-MS (DTIMS-MS) platform and trapped IMS-MS (TIMS-MS) instrument to further characterize the unfolding of bovine SOD1 by evaluating solution based changes and occurrence of the single-metal monomer. To assess the unfolding of SOD1, different solution conditions with increasing concentrations of acid were used to induce systematic denaturation of the SOD1 holo-dimer into its associated oligomers. This approach is commonly employed in other biophysical techniques, including spectroscopic analyses and NMR, to investigate multiple protein folding states. Thus, one of the primary goals of this experiment was to use a similar approach in order to make CCS measurements of the SOD1 holo-dimer and its associated oligomers that could be comparable to those investigated using other biophysical techniques. In using different solvent conditions, direct measurements of the CCS of the SOD1 holo-dimer, holo-monomer, single-metal monomer, and apo-monomer in their associated solutions were performed without the need for instrument modification beyond the base model, such as those needed for CIU. The DTIMS-MS platform was used to evaluate SOD1 in the various solution conditions. The unfolding of the holo-dimer was then examined, and CCS values were reported for the holo-dimer, holo-monomer, single-metal monomer, and apo-monomer. Results from the DTIMS-MS and TIMS-MS platforms were then compared with CCS measurements predicted from bovine SOD1 crystal and NMR structures to assess the similarity of the CCS measurements and other biophysical methods.

## Materials and Methods

### Materials

Lyophilized bovine superoxide dismutase 1 (SOD1) from erythrocytes (S7446-15KU) was purchased from Sigma Aldrich (Burlington, MA) and reconstituted in 20 mM ammonium acetate to a concentration of 40 µM SOD1 without further modification. LC-MS grade water, acetonitrile (ACN), ammonium acetate, and formic acid (FA) were purchased from Fisher Scientific (Hampton, NH) and used as received.

### Sample Preparation

Initially, the SOD1 protein concentration was determined by measuring the characteristic absorbance at 258 nm produced by the Cu in the enzyme active site, with an extinction coefficient of 10,300 M^−1^ cm^−1^ (McCord and Fridovich, 1969). Metal content was measured by inductively coupled plasma-mass spectrometry (ICP-MS, 7700s Agilent) by mixing 20 µL of 65.1 µM SOD with 480 µL of 1% nitric acid. The metal to protein molar ratios were: 0.86 ± 0.1 Cu:protein and 0.88 ± 0.1 Zn:protein. To prevent the possibility of cosolute formation at the flow rate used for the TIMS-MS experiments, no additional Cu^2+^ and Zn^2+^ were added to the sample solution conditions. For the native condition experiments, the reconstituted SOD1 stock solution was diluted to a final concentration of 20 µM in 30 mM ammonium acetate buffer. To ensure that the SOD1 holo-monomer was present, a second SOD1 solution was prepared to a final concentration of 20 µM in 30% ACN with 100 µM FA. Formation of the SOD1 apo-monomer was promoted in a third sample condition by diluting SOD1 to 20 µM in 30% ACN with 25 mM FA. All solutions were analyzed immediately after preparation to minimize potential protein denaturation.

### DTIMS-MS Analyses

For the DTIMS-MS measurements, the SOD1 solutions were directly infused into the Agilent nanospray ion source (Santa Clara, CA) at a rate of 300 nL/min via syringe pump (Harvard Apparatus PHD 2000; Holliston, MA) and ionized at 1500 V. The source was interfaced with the Agilent 6560 IMS-QTOF MS (Santa Clara, CA) outfitted with a commercial gas kit (Alternate Gas Kit, Agilent, Santa Clara CA) and a precision flow controller (640B, MKS Instruments, Andover, MA). Instrumental parameters were selected based on a previously published method enabling standardized collision cross section (CCS) measurements (Stow et al., 2017). In this method, the IMS separation was performed using nitrogen drift gas maintained at a constant pressure of 3.95 Torr and a uniform single electric field of 17.3 V/cm. Mass spectrometry data was acquired in positive ionization mode from 100 to 20,000 *m/z*. A detailed description of source parameters, IMS parameters, and TOF settings is given in Supplementary Tables S1–S3.

The SOD1 holo-dimer, holo-monomer, single metal-monomer and apo-monomer CCS values were determined using the single-field method (Stow et al., 2017). In this approach, a series of calibrant tune mix ions (Agilent tune mix) is first analyzed and their respective drift times measured under experimental conditions identical to those utilized for the protein samples. These tune mix ions have been highly characterized and possess well-defined CCS values. Drift times for the tune mix ions and the SOD protein samples were analyzed utilizing the Agilent IM-MS Browser version 10.0 (Santa Clara, CA). A linear regression derived from the Mason-Schamp equation (Eq. 1) (Stow et al., 2017)

$$t_{A}=\;\frac{\beta }{z}\;{\left[\frac{m_{i}}{m_{\beta }+m_{i}}\right]}^{1/2}\mathrm{CCS}+t_{\text{fix}},$$ (1)

was then used to generate a calibration line correlating observed tune mix ion drift times with their CCS values. The resulting calibration line was then used to calculate the CCS values for the SOD1 holo-dimer, holo-monomer, single metal-monomer and apo-monomer. Theoretical CCS values for each SOD complex were then determined using the Ion Mobility Projection Approximation Calculation Tool (IMPACT) algorithm (Marklund et al., 2015) for comparison with the experimental CCS values.

### TIMS-MS Analyses

The SOD1 solution was introduced by direct infusion with a syringe pump at 1.5 μL/min using an Apollo II Standard ESI Source (Bruker) with nanoBooster (ACN) used to increase the ionization efficiency by reducing the interfacial force of the droplets formed by electrospray. The mobility scan range was 0.95–1.65 V*s*(cm^2^)^−1^ with an accumulation time of 250 ms (100% duty cycle), with the IMS funnel 1 RF set to 350 Vpp. Capillary voltage was set to 4500 V, dry gas 2.6 L/min, and dry temperature set to 180 °C. The mass range analyzed in positive ionization mode ranged from 550 to 6,000 *m/z*. A detailed description of source and TIMS settings can be found in the Supplementary Figures S1-S6.

## Results and Discussion

IMS-MS is a powerful technique for the comprehensive elucidation of protein folding and dynamics in circumstances where other biophysical methods, such as cryo-EM or crystallography, fail to detect intermediates, subtle changes, or cannot perform due to low concentrations. In its correctly folded form, SOD1 exists as a dimer with both a Zn and Cu metal ion bound to each monomer subunit (Lynch et al., 2004; Ding and Dokholyan, 2008; Kayatekin et al., 2008). The loss of one or both metal ions is however thought to lead to instability of the protein’s secondary structure, but currently only incomplete or artificial structures exist from both NMR and X-ray crystallography for these circumstances. Since it has been illustrated that acidic (Goto et al., 1993; Konermann et al., 1997) or high organic environments (Partridge et al., 1999) can be used to denature proteins from their native states, we utilized three different solvent systems to investigate SOD1 intermediates occurring from the holo-dimer to apo-monomer forms. The CCS values for each dimer and monomer complex were investigated in the various solutions to determine the relationship between metal cofactors and structural stability.

### SOD1 Nested DTIMS-MS Distributions

**FIGURE 1:**
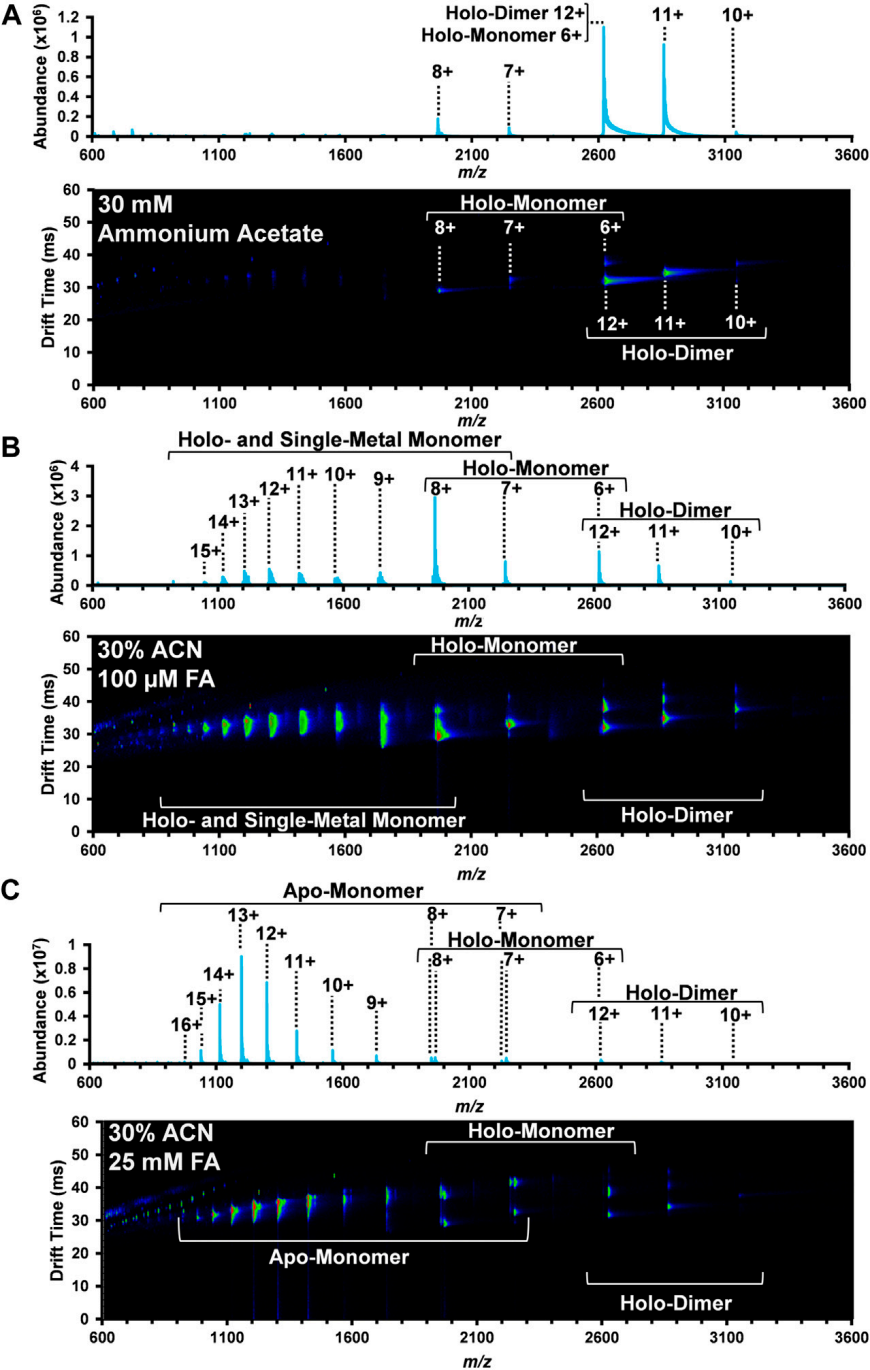
Nested DTIMS-MS spectra for bovine SOD1 in **(A)** 30 mM ammonium acetate; **(B)** 30% ACN, 100 µM FA and 30 mM ammonium acetate; and **(C)** 30% ACN, 25 mM FA and 30 mM ammonium acetate. In the native solution condition **(A)**, SOD1 primarily existed in its native holo-dimer form. Addition of ACN and FA, however, led to the formation of an extended holo-dimer **(B–C)**, holo-monomer **(B–C)**, single-metal monomer **(B)**, and the apo-monomer **(C)**.

The use of multiple solvent systems to probe the unfolding dynamics of proteins, either through the use of acidic conditions (Goto et al., 1993; Konermann et al., 1997), chaotropic reagents like guanidine hydrochloride (Greene and Pace, 1974; Mei et al., 1992; Huerta-Viga and Woutersen, 2013), or the addition of organic solvents (Partridge et al., 1999) has been a common practice for biophysical investigations. In this study, the bovine SOD1 holo-dimer was initially investigated in 30 mM ammonium acetate, as this solvent system is known to best preserve the native conformation of protein complexes. ACN and FA were then utilized for systematic denaturation of the protein complexes. Figure 1 illustrates the nested DTIMS drift time and *m/z* spectra for SOD1 in three different conditions: 30 mM ammonium acetate (A), 30% ACN, 100 µM FA and 30 mM ammonium acetate (B), and 30% ACN, 25 mM FA solution and 30 mM ammonium acetate (C). The exact *m/z* detected for each form of SOD1and the corresponding single-field ^DT^CCS_N2_ are included in the Supplementary Material. The ACN and FA concentrations were selected based on previous work that demonstrated 25 mM FA promoted the formation of the apo-monomer (Forman and Fridovich, 1973; Hayward et al., 2002), while a less acidic solution was previously used to investigate the SOD1 holo-monomer (Rhoads et al., 2011).

In the native solution (Figure 1A), the mass spectrum obtained from the DTIMS platform is predominately comprised of the SOD1 holo-dimer with a small amount of holo-monomer. Peaks at 3,144, 2,858, and 2,620 *m/z* were the most abundant and correlate with the 10+, 11+, and 12+ charge states of the holo-dimer. Interestingly, overlapping isotopic distributions were observed at 2,620 *m/z*, but the IMS separation allowed for discrimination between the 12+ holo-dimer and 6+ holo-monomer due to their drastically different sizes and charge migration through the drift cell. Other SOD1 holo-monomers were also observed at 2,246 and 1965 *m/z*, corresponding to the 7+ and 8+ charge states. As proteins in their native form are limited in their amount of surface charge, the more monomodal charge state distribution is consistent with a more compact, native protein structure (Dobo and Kaltashov, 2001; Testa et al., 2011; Hall and Robinson, 2012; Li et al., 2017). Furthermore, the observed range of charge states for the holo-dimer is consistent with those previously reported for native MS experiments involving SOD1 analyses (Borges-Alvarez et al., 2013; Zhuang et al., 2014; Li et al., 2017).

The second solvent system utilized for the DTIMS assessment was 30% organic solvent (ACN) and 100 µM FA in 30 mM ammonium acetate to promote the denaturation of the SOD1 holo-dimer. This solvent system has previously been used for the direct analysis of SOD1 from spinal cord tissue (Rhoads et al., 2011) and was demonstrated to promote the formation of the monomeric form of SOD1. As in the native solution condition, the holo-dimer was observed from the 10+ through 12+ charge states (3,144, 2,858, and 2,620 *m/z*, respectively), but an increased abundance of SOD1 holo-monomer also occurred (Figure 1B). Here, the nested DTIMS-MS spectrum revealed both an extended and compact form for the 11+ holo-dimer (Figure 1B), in contrast to the native solution which only had a compact form. Similar to the native solution, the holo-monomer was again present for the 6+ through 8+ charge states (2,620, 2,246, and 1965 *m/z*, respectively), but additional peaks were observed at lower *m/z*, corresponding to higher charge states of the holo-monomer (see Supplementary Material for exact *m/z* values). Since higher charge states are typically associated with a more denatured state, these solution conditions appeared to greatly affect the protein structure (Kaltashov and Mohimen, 2005). The single-metal form of the holo-monomer was also detected in this solution condition (Supplementary Figure S7), however the missing metal co-factor could not be readily determined (see Supplementary Material for exact *m/z* values). The masses of ^63^Cu and ^64^Zn differ by only 0.999545 Da (Rhoads et al., 2013) and have overlapping isotopic distributions. Furthermore, large proteins often exhibit a broad isotopic distribution due to the natural abundance of ^13^C and other isotopes, making the assessment of metal amounts for copper and zinc exceedingly difficult (Rhoads et al., 2011; Xian et al., 2012; Rhoads et al., 2013; Adams et al., 2018). This was previously observed in single-metal SOD1 measured from the spinal cord tissue of transgenic mice where the authors were also unable to distinguish between copper-containing, zinc-deficient SOD1 and the zinc-containing, copper-deficient form of SOD1 due to the overlap of the protein isotopic distribution (Rhoads et al., 2011).

The highest FA concentration (25 mM FA) with 30% ACN and 30 mM ammonium acetate was then used to further denature SOD1 as shown in Figure 1C. This solvent system has previously been used to perturb the protein structure to promote the formation of the apo-form of SOD1 (Hayward et al., 2002). As shown in Figure 1C, this solution condition created the largest disturbance to the native SOD1 structure as the holo-dimer was the least abundant protein form detected. The holo-dimer was however still observed in the 10+ through 12+ charge states, similar to the native conditions, and the 11+ holo-dimer had both a compact and extended form, comparable to the 30% ACN and 100 µM FA condition (Figure 1C). While the holo-monomer was present from the 6+ through 8+ charge states, this solution was dominated by the formation of the apo-monomer, which also had the greatest charge state range of all conditions (7+ through 16+, exact *m/z* are detailed in the Supplementary Material). As previous research has correlated increases in charge state to greater surface area, this solution appears to favor the most elongated, denatured version of the protein (Kaltashov and Mohimen, 2005; Testa et al., 2011).

### DTIMS Analysis of the SOD1 Holo-Dimer Complexes

**FIGURE 2:**
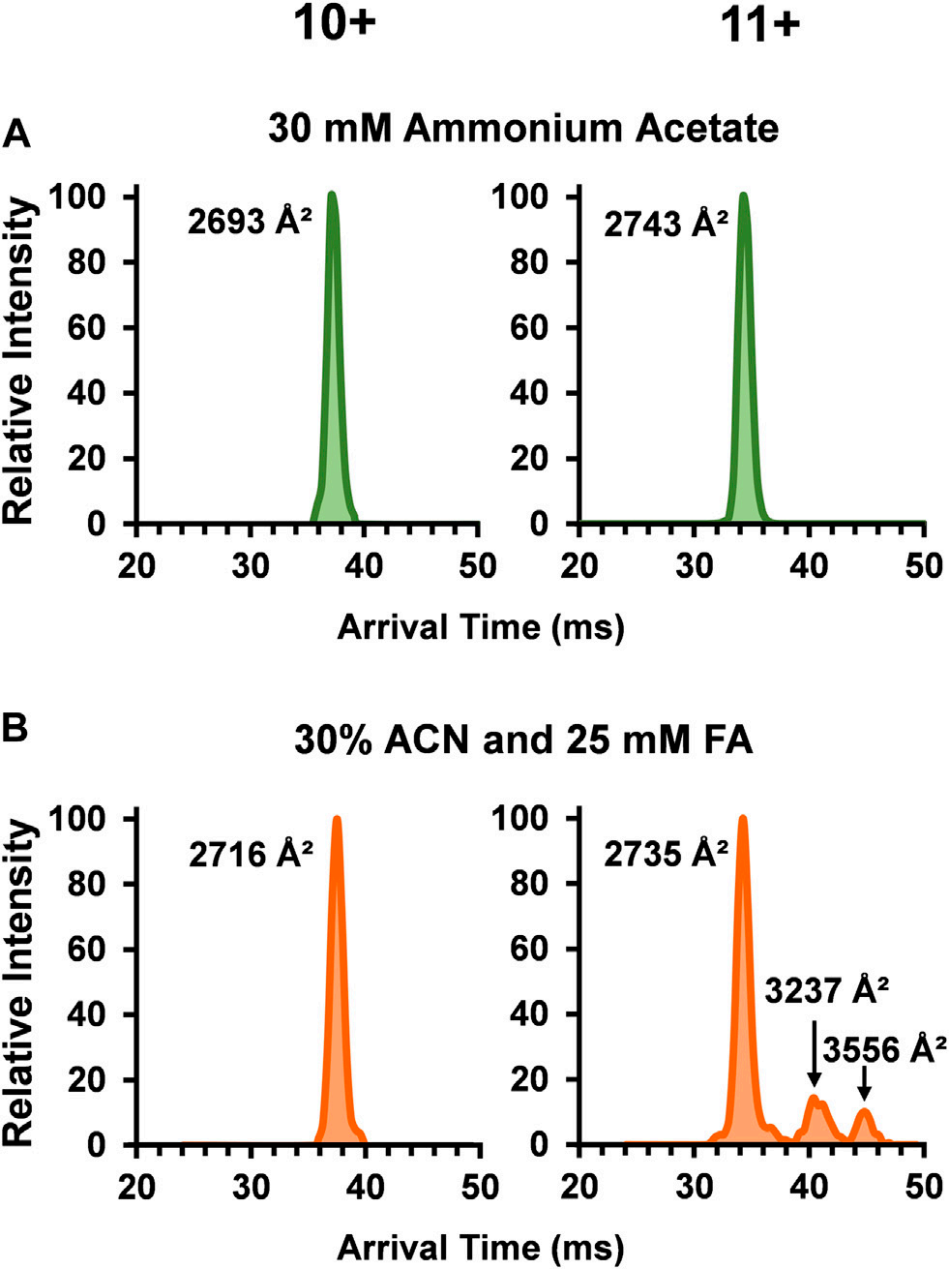
Arrival time distributions (ATD) for the SOD1 holo-dimer in **(A)** 30 mM ammonium acetate and **(B)** 30% ACN, 25 mM FA and 30 mM ammonium acetate. The native condition holo-dimer displayed a compact, monomodal ATD for both the 10+ and 11+ charge states. In the harshest solution condition applied **(B)**, the observed ^DT^CCS_N2_ of the 10+ charge state holo-dimer slightly increased and the 11+ charge state showed three monomodal conformations where one was similar to that observed in **(A)**, and the two additional structures had a larger ^DT^CCS_N2_ values, implying the formation of extended holo-dimers.

To unveil more in-depth structural information for SOD1, ^DT^CCS_N2_ values for each dimer peak in the three solvent systems were assessed with DTIMS (see Supplementary Material for single-field CCS calculations and associated % RSD). While the SOD1 holo-dimer was detected in all three of the solution conditions, its abundance greatly decreased as organic and acid amounts increased. Examination of the arrival time distribution (ATD) for the holo-dimer in the native solution (30 mM ammonium acetate) showed a compact, monomodal ATD for the 10+ charge state with a ^DT^CCS_N2_ of 2,693 Å^2^ (Figure 2A). A slightly larger ^DT^CCS_N2_ value of 2,743 Å^2^ was noted for the 11+ charge state, consistent with previous IMS-MS investigations of proteins as the increased charge state causes expansion of the protein due to charge repulsion. While the 10+ charge state maintained its compact, monomodal distribution even in the harshest solution condition (30% ACN and 25 mM FA), the ^DT^CCS_N2_value increased to 2,716 Å^2^ or ∼1% larger than that observed in the native solution, illustrating a slight amount of extension. The 11+ holo-dimer ATD was however strikingly different in the harshest solution condition as three conformations were present (Figure 2B). Here, the smallest structure was similar to the native form (2,735 Å^2^), but two new extended forms (3,237 Å^2^ and 3,556 Å^2^) arose. While IMS-MS has not previously been used to investigate the impact of acidic and organic solutions of SOD1, the unfolding pathway of the SOD1 holo-dimer has been studied using CIU and traveling wave IMS-MS (TWIMS-MS). In this study, a similar phenomenon in the 11+ holo-dimer in the native solution was observed upon increasing the sample cone voltages (Zhuang et al., 2014).

### DTIMS-MS Analyses of SOD1 Monomers

**FIGURE 3:**
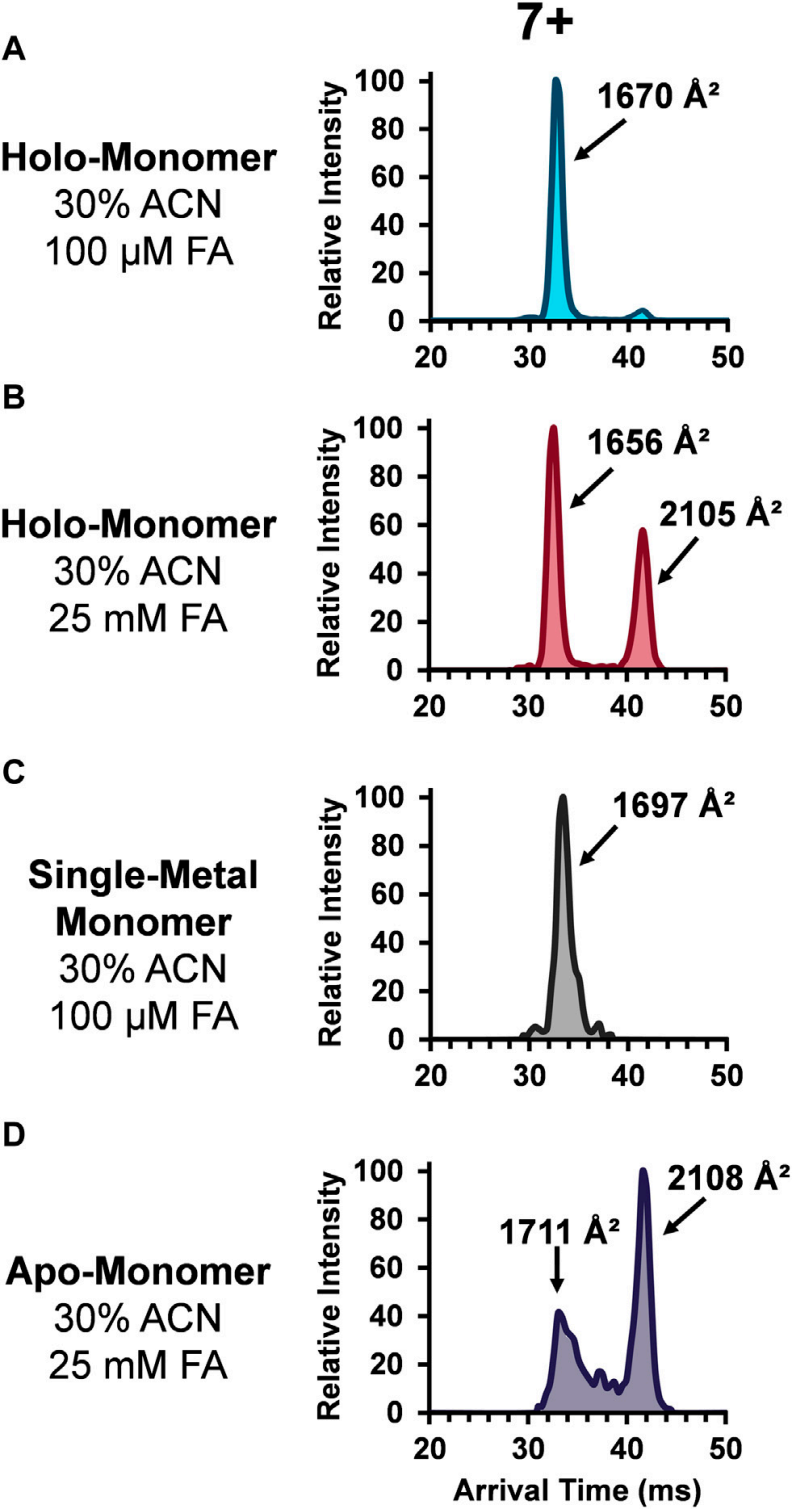
ATDs for the SOD1 holo-monomer in **(A)** 30% ACN, 100 µM FA and 30 mM ammonium acetate and **(B)** 30% ACN, 25 mM FA and 30 mM ammonium acetate, as well as the single-metal monomer **(C)** and the SOD1 apo-monomer **(D)**.

The role of metal ligands on SOD1 enzymatic activity and structural stability has also been of great interest. Primarily, the copper ion in each SOD1 subunit is responsible for the catalytic activity, while the closely coordinated zinc ion maintains its structure. Additionally, the thermodynamic (Lindberg et al., 2002; Stathopulos et al., 2003) and kinetic stability (Lynch et al., 2004) of SOD1 is linked with its metal ligands. Thus, understanding the role these metal ions play in contributing to the protein tertiary and quaternary structure of SOD1 is integral to understanding their biological implications. Given the importance of the metal cofactors in stabilizing SOD and the fact the abundance of the monomers increased upon the addition of organic and acidic solvent, the ATDs for the holo-monomer, single-metal monomer, and apo-monomer were assessed. Figure 3 depicts the lowest charge state (7+) of the different SOD1 monomers detected. The SOD1 holo-monomer was observed in both the 30% ACN and 100 µM FA (Figure 3A) and the 30% ACN and 25 mM FA solutions (Figure 3B). Since the holo-monomer of SOD1 contains one Cu^2+^ and Zn^2+^ ion per monomeric unit, it was anticipated it would primarily be in its compact form. This was the case for the 30% ACN and 100 µM FA solution, where the holo-monomer was observed to primarily be in its compact form with a calculated ^DT^CCS_N2_ of 1,670 Å^2^. However, increasing the concentration of FA to 25 mM resulted in a difference in both the ^DT^CCS_N2_ and ATD for the same charge state (Figure 3B). In this condition, the 7+ holo-monomer exhibited a slight compaction in structure and a prominent extended structure with ^DT^CCS_N2_ values of 1,656 Å^2^ and 2,105 Å^2^. This extension of the holo-monomer in the higher acid condition implies further denaturation of the holo-dimer in this solution condition. Additionally, the 100 µM FA and 30% ACN also promoted formation of the single-metal SOD1 monomer, but as noted previously, the mass resolution of the TOF mass spectrometer was insufficient for determining which metal cofactor was lost (Rhoads et al., 2011; Rhoads et al., 2013), so the ATDs and ^DT^CCS_N2_ values have been labeled ambiguously. However, to determine the impact of the single metal cofactor loss on the subunit’s tertiary structure, the ^DT^CCS_N2_ and ATDs of the holo-monomer and single-metal monomer were compared at the lowest charge state observed for both forms. Similar to the holo-monomer, the ATD of the 7+ charge state of the single-metal monomer displayed a compact conformation with a ^DT^CCS_N2_ of 1,697 Å^2^ (Figure 3C), which was 2% larger than that of the holo-monomer (1,670 Å^2^) in the same solution. As previously mentioned, addition of 30% ACN and 25 mM FA promotes the formation of the apo-monomer. When the equivalent charge state and solution of the SOD1 holo-monomer and apo-monomer were compared, the apo-monomer had a larger ^DT^CCS_N2_ for both its extended and compact form, and the extended form was the predominant conformer present (Figure 3D). Furthermore, the apo-monomer ATD failed to return to baseline between the two conformations, indicating the presence of intermediate structures (Figure 3D).

Instability resulting from different degrees of metalation, demonstrated by these data, are in strong agreement with information that has been garnered using other biophysical techniques (Lynch et al., 2004; Roberts et al., 2007; Nordlund and Oliveberg, 2008; Nedd et al., 2014). However, one of the major advantages of using IMS-MS to characterize protein structure is the ability to examine multiple protein states simultaneously, a capability that precludes X-ray crystallographic or NMR analyses. Previous examination of the kinetics of acid-induced unfolding of SOD1 using fluorescence demonstrated that the apo-protein displays a higher unfolding rate than the holo-protein and derives structural stability from the presence of its metal cofactors (Lynch et al., 2004). In this study by Lynch and co-workers (Lynch et al., 2004), the authors observed a series of unfolding phases, particularly for the SOD1 apo-monomer. This is consistent with the ATDs observed for the apo-monomer, which have apparent intermediate conformers between the compact and extended conformations. The presence of metals within the monomer, however, led to the observation of monomodal ATDs for the SOD1 monomer at the lowest observable charge state. This finding has been corroborated by several additional studies, which indicate that the metal cofactors in SOD1 play an important role in maintaining the protein structure (Roberts et al., 2007; Nordlund and Oliveberg, 2008). Indeed, while Zn^2+^ appears to be the cofactor most responsible for maintenance of protein structure, the loss of a single metal cofactor has not been demonstrated to cause complete failure of protein tertiary structure (Roberts et al., 2007; Nedd et al., 2014). Thus, our findings of a single-metal SOD1 monomer with an almost monomodal ATD is consistent.

**FIGURE 4:**
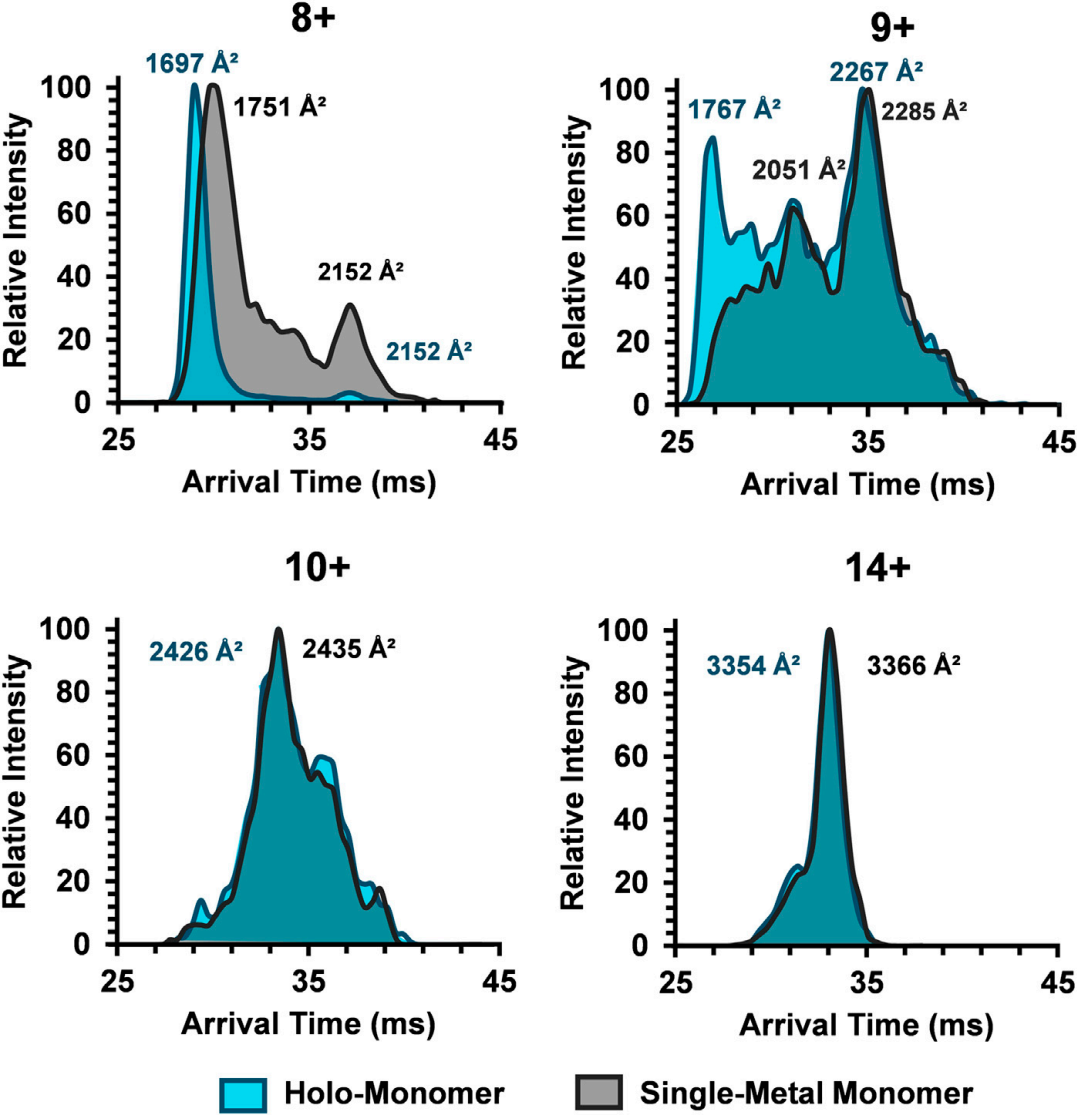
Comparison of the SOD1 ATDs for the holo-monomer and single-metal monomer for the 8+, 9+, 10+, and 14+ charge states in the 30% ACN, 100 µM FA and 30 mM ammonium acetate. In the 8+ charge state, the compact holo-monomer was slightly smaller than the single-metal monomer, while the extended states had similar ^DT^CCS_N2_ values. As the charge state increased, the ATDs of the holo-monomer and single-metal monomer began to overlap, both showing similar extension.

To better characterize the relationship between metalation and charge state on the SOD1 subunits, ATDs for the holo-monomer and single-metal monomer were overlaid and discrepancies between the two were explored (Figure 4). The discrepancies in ATDs between the holo-monomer and single-metal monomer at the lower observed charge states, such as the 8+ charge state, reveal differences between the two protein forms. At the 8+ charge state, the holo-monomer had a monomodal ATD primarily comprised of a compact monomer, while the single-metal monomer displayed both a substantial compact and extended form, each with a higher arrival time than the holo-monomer. As the charge state increased to 9+, the holo-monomer also exhibited both a compact and extended form with ^DT^CCS_N2_ values of 1767 Å^2^ and 2,267 Å^2^. Additionally, the ATD for the holo-monomer did not return to baseline, indicating intermediates between the compact and extended SOD1 holo-monomer (Figure 4). The single-metal monomer had a similar ATD at this charge state, showcasing a compact and extended conformation and numerous intermediates. Ultimately as the charge state increased, the holo- and single-metal monomer began to converge. As seen in Figure 4, the ATD for the 10+ charge state shifts to a more appreciable overlap for the two forms of SOD1 and remained true for the remainder of the higher charge states. An example of this increased overlap can be seen in the 14+ charge state where the ^DT^CCS_N2_ values for the holo-monomer and the single-metal SOD1 monomer were within instrumental error of each other (∼0.3%). Furthermore, the charge state repulsion at the higher charge states appears to overcome any structural benefit that the metal cofactor may attribute to the structure of the single-metal or holo-monomer, allowing the protein to elongate into its extended conformation regardless of the degree of metalation.

**FIGURE 5:**
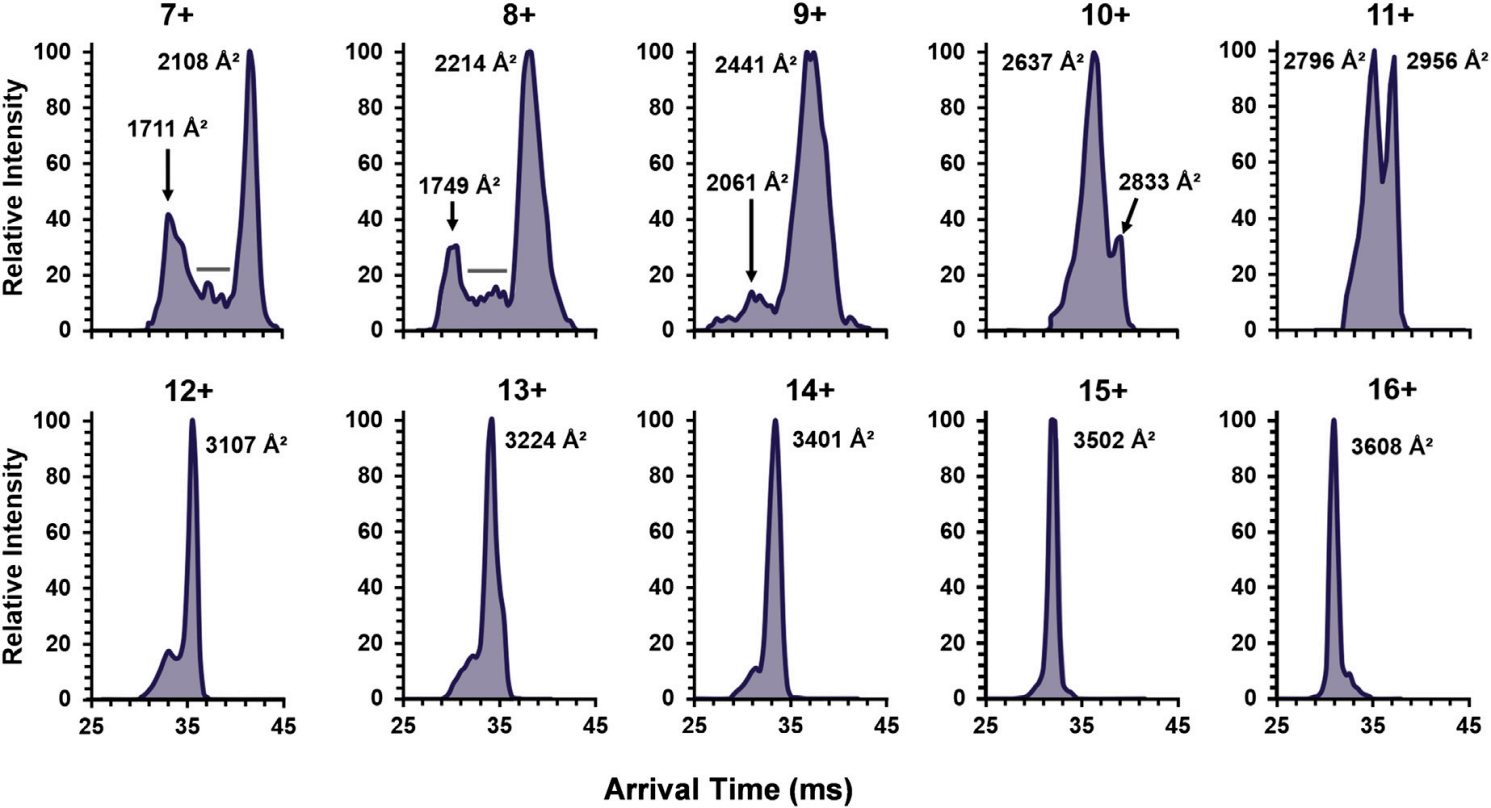
The ATDs for the SOD1 apo-monomer observed in 30% ACN with 25 mM FA for the detected charge state range of 7+ to 16+. The lower charge states revealed both extended and compact structures with evidence for intermediates (denoted by the gray bars). Furthermore, after the 10+ charge state, monomodal extended conformers were observed.

To continue examination of metal effects, the increasing charge state ATDs of the apo-monomer were examined (Figure 5), revealing similar results to the single-metal monomer and holo-monomer (Figure 4). At the lower charge states (7+ and 8+) for the apo-monomer, there is a clear differentiation between the compact and extended form with intermediate conformations between the two distributions, as denoted by the gray bar (Figure 5). As the charge state increase, apo-monomer exhibited equal abundance between the compact and extended conformations (11+) and then a shift to the extended conformation (>11+), where at the 16+ charge state the most extended state of the apo-monomer measured 3,608 Å^2^ was observed.

### Unfolding Pathway for SOD1

**FIGURE 6:**
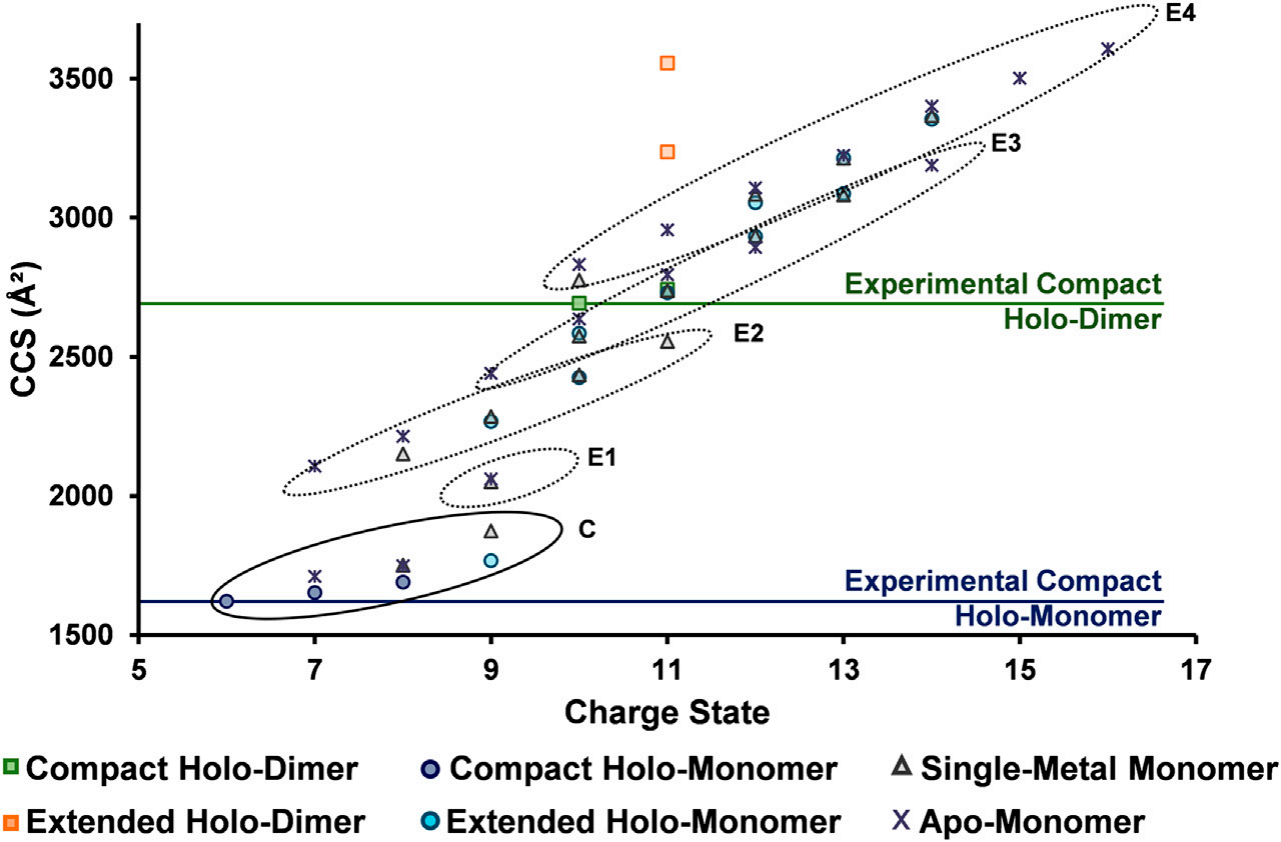
^DT^CCS_N2_ vs. charge state trends for the SOD1 complexes illustrating its unfolding regimes. The lowest observed charge states (considered to be the compact form) for the holo-dimer and holo-monomer are denoted by horizontal lines and extension are illustrated by the different circled regions. For the SOD1 monomer, four separate elongation regions were observed as charge state increased (E1 through E4), indicating elongation is a stepwise process. The extended holo-dimer ^DT^CCS_N2_ values also showed that upon denaturation, it can exist in two other forms.

To fully understand the unfolding intermediates and pathway occurring in SOD1, experimental ^DT^CCS_N2_ values obtained with the differing solution conditions and charge states were plotted for all SOD1 complexes (Figure 6). The lowest charge state observed for the SOD1 holo-dimer (10+) was attributed to be its native, compact form (horizontal line denoted in Figure 6). However, upon denaturing conditions, the 11+ charge state departed from the compact form as denoted by the orange boxes (Figure 6). Trends in the monomer were then examined. As anticipated, the lowest observed charge state for the holo-monomer (6+, denoted by the horizontal line in Figure 6) had the smallest observable ^DT^CCS_N2_. However, upon denaturation and increase in charge state, four separate elongation regions were observed for the holo-monomer (noted as E1 through E4 in Figure 6). This trend began at the 9+ charge state, where a shift away from the compact form of the SOD1 and first elongation region of SOD1 was observed. Additionally, while both the single-metal and apo-monomer were observed from the 7+ through 9+ charge states in both an extended and compact form, their extended form fell within the second elongation region of the monomer. As the charge state continued to increase, the ^DT^CCS_N2_ of the holo-monomer, single-metal monomer, and apo-monomers also increased. As observed in the ATDs for the single-metal and holo-monomer in Figure 4, after the 10+ charge state, the ATDs for the different monomers begin to coincide, and the difference in the ^DT^CCS_N2_ for the two was within instrumental error. At this point, it appears that elongation of the monomers is primarily driven by Coulombic repulsion in the gas phase. The largest observed ^DT^CCS_N2_ was the 16+ apo-monomer with a ^DT^CCS_N2_ of 3,608 Å^2^. This form of the SOD1 apo-monomer is the most unfolded conformation of the SOD1 monomer most likely due to both gas-phase Coulombic repulsion and the lack of metal cofactors. These experimental ^DT^CCS_N2_ values may serve as a starting point for molecular dynamics simulations to further elucidate the structural changes associated with the increasing charge states.

**FIGURE 7:**
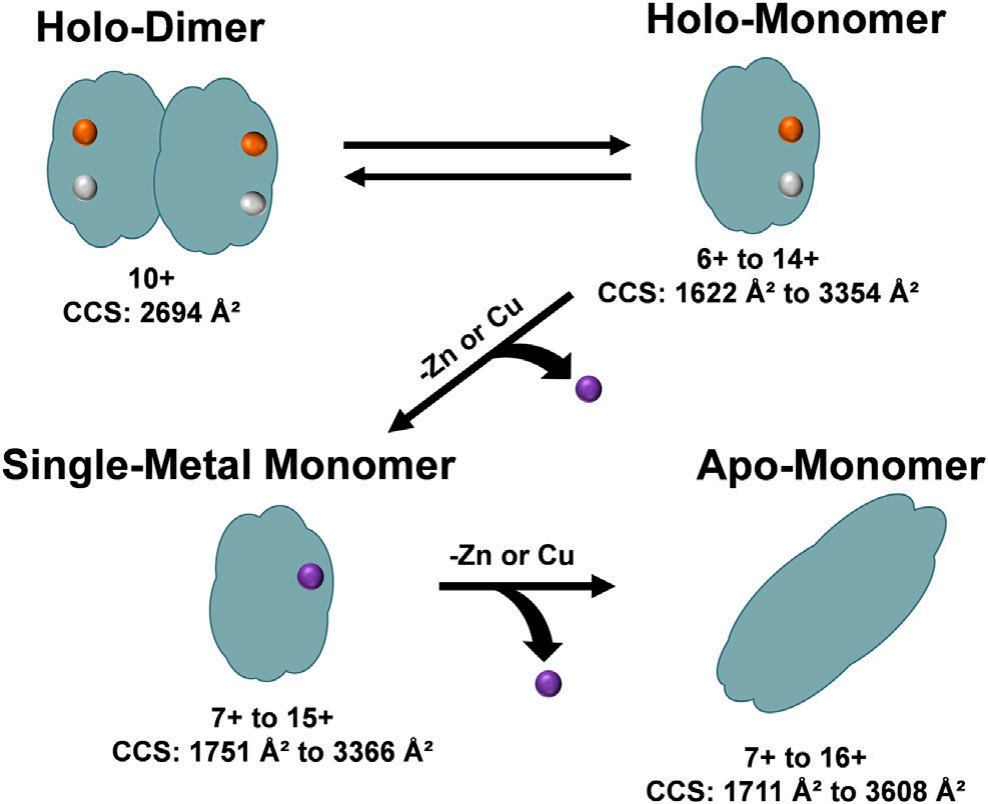
An overview of the unfolding pathway of SOD1 as observed from the DTIMS ^DT^CCS_N2_ data. The native structure of SOD1 is a holo-dimer comprised of two monomers, each containing one Zn^2+^ and one Cu^2+^ ion. After addition of acid to the solution, the SOD1 holo-dimer elongated and dissociated into holo-monomers. The holo-monomers were further denatured from their native state by loss of a metal ligand, leading to the formation of a single-metal monomer. Further loss of the additional metal ion led to the formation of the apo-monomer, which extended to the largest ^DT^CCS_N2_ values observed for all protein structures.

The unfolding data observed in Figure 6 and order of appearance of the different oligomers within the solution conditions (Figures 1A–C), allowed for the elucidation of a general unfolding pathway of SOD1 (Figure 7) when these solution conditions are used. This pathway begins with the SOD1 holo-dimer, which is the most abundant in native solution condition. Elongation of the holo-dimer was then observed at the 11+ charge state and dissociation of the dimer into monomeric SOD1 units as the solution was modified from native conditions. In the intermediate pH and organic condition (30% ACN with 100 µM FA), an increased amount of the holo-monomer was observed, along with the formation of the single-metal monomer. Harsher solution conditions (additional FA) then promoted the formation of the apo-monomer. The unfolding pathway proposed here is consistent with other research regarding unfolding of the SOD1 holo-dimer, which proposes a multi-step denaturation pathway for the protein (Arnesano et al., 2004; Doucette et al., 2004; Ray et al., 2004). The dissociation of the holo-dimer is shortly followed by the loss of the metal cofactors, which further destabilized the protein monomers, ultimately resulting in formation of the apo-protein (Mei et al., 1992; Assfalg et al., 2003; Arnesano et al., 2004). It is important to note that under other solution conditions, additional means of dissociation and elongation of this protein may be attained. Thus, while this data demonstrates a potential unfolding pathway for the SOD1 holo-dimer, it is possible that the unfolding pathway is solution-dependent. Regardless, the ability to directly measure the intermediate cross section may serve to aid in the elucidation of the structural changes that contribute to disease states such as ALS. Furthermore, several of the forms observed in this unfolding pathway have previously been measured directly from the spinal cord tissue of transgenic mice using MS (Rhoads et al., 2011). In particular, the metal-deficient forms are of great interest as this state is thought to be the unstable intermediate that results in the toxicity observed in ALS (Rhoads et al., 2011; Hilton et al., 2015).

### Comparison of CCS Values Using DTIMS-MS and TIMS-MS

Since this application shows the great utility of IMS for protein conformational analyses, two IMS techniques, the Agilent DTIMS and Bruker TIMS platforms were utilized to examine SOD1 in native conditions (30 mM ammonium acetate). The use of DTIMS to measure the CCS of large molecular complexes has a rich foundation in scientific literature including the investigation of the native structure of numerous proteins such as ubiquitin (Wyttenbach and Bowers, 2011), amyloid-β (Wyttenbach et al., 2014), cytochrome C (Clemmer et al., 1995; Shelimov et al., 1997; May et al., 2018), and myoglobin (May et al., 2018). In stark contrast, the application of TIMS instruments for the analysis of native proteins is rather recent. This technique provides unique challenges for native MS analyses, as it depends upon rf confinement to trap ions, decoupling their mobility from the time domain (Ridgeway et al., 2018). The use of rf confinement lends to a higher potential for rf heating of ions to occur, which could alter the conformation of proteins from their native state (Benigni et al., 2016). Thus, in this study parameters for the Bruker TIMS were carefully selected such that rf heating of SOD1 and associated oligomers was minimized. Representative spectra for the data obtained from the TIMS-MS measurements are shown in Supplementary Figures S8-S9. Upon comparison of the CCS values both instruments showed excellent correlation with one another. In the assessment of the 7+ holo-monomer in 30 mM ammonium acetate, DTIMS reported a ^DT^CCS_N2_ value of 1,653 Å^2^, while TIMS was only slightly larger (0.7%), measuring 1,663.5 Å^2^. The CCS for the 10+ SOD1 holo-dimer using DTIMS was 2,694 Å^2^ and 2,629 Å^2^ with TIMS, 2.4% smaller. Experimental values from both the DTIMS and TIMS platforms were then compared with computational CCS values calculated from the SOD crystal structure using IMPACT (Marklund et al., 2015). IMPACT is an algorithm that more rapidly calculates CCS form protein structures compared to other approaches such as MobCal, making it an attractive option for structural proteomics studies. IMPACT can provide theoretical CCS values using both the projection approximation and the trajectory method. For these experiments, the standard settings for IMPACT were used to calculate theoretical CCS values based on NMR and X-ray crystal structures obtained from the PDB, and the resulting trajectory method predicted CCS values were compared to experimentally-obtained single-field CCS measurements. In order to assess the relationship between our experimental values and those obtained from IMPACT, it was necessary to first examine the native SOD1 holo-dimer. As the holo-dimer is the native form of bovine SOD1, both NMR and crystallography data regarding the protein structure is available from the Protein Data Bank (PDB) (https://www.rsbc.org) (Berman et al., 2000). The structures used for computational CCS calculations were PDB ID: 1E9P for the crystallography data (Hough et al., 2000), and 1L3N for NMR comparison (Banci et al., 2002). The experimental and theoretical values for the CCS of the SOD1 holo-dimer at its lowest observed charge state are shown in Table 1. Trajectory method (TJM) results for the 10+ SOD1 holo-dimer using IMPACT predicted a CCS of 2,701 Å^2^ when using the crystal structure (PDB ID: 1E9P) for CCS prediction. In comparison with experimental results from the DTIMS platform, the theoretical CCS differed by 0.3%, while the TIMS CCS value was 2.7% smaller. The CCS predicted from the NMR structure was slightly larger than that predicted by the crystallography data, measuring at 2,722 Å^2^. This showed similar agreement with the DTIMS and TIMS experimental data, which differed by 1 and 3.5% respectively. However, it is worth noting that the SOD1 holo-dimer NMR structure used for this calculation was actually an average of several structures that make up the ensemble of structures that fit the NMR data, thus the higher difference between the experimental and computational CCS values may be attributed to poorly constrained structures. Similarly, there is a NMR structure of the apo-state of SOD1 (PDB ID: 1RK7) (Banci et al., 2003), which was demonstrated to have a 2.3% difference with the measured structure from the DTIMS platform, however since only the native solution measurements were done with TIMS, no apo-monomer CCS value was available for comparison.

**TABLE 1:** Comparison of Computational CCS values predicted crystallography and NMR data with experimental data from the DTIMS and TIMS platforms for the SOD1 holo-dimer. Computational CCS values were determined using the trajectory method (TJM) using IMPACT.

**Holo-Dimer**						
PDB structure	Analytical method	Calculated CCS-TJM^a^	Measured ^TIMS^CCS_N2_ (Å^2^)	% Diff TJM	Measured ^DT^CCS_N2_ (Å^2^)^b^	% Diff TJM
1E9P	Crystallography	2,701 (9)	2,629 (1)	2.7	2,693.5 (5)	0.3
1L3N	NMR	2,722 (20)	2,629 (1)	3.5	2,693.5 (5)	1.0
Apo-monomer						
PDB structure	Analytical method	Calculated CCS- TJM a	Measured TIMSCCSN2 (Å^2^)	% Diff TJM	Measured DTCCSN2 (Å^2^)b	% Diff TJM
1RK7	NMR	1751 (26)	N.D.	N.D	1711 (5)	2.3

^a^ Calculated using IMPACT algorithm (Marklund et al., 2015).

^b^ Mean (standard deviation) from *n* = 3 measurements.

## Conclusion

In this study, IMS-MS was utilized to study both the structure and unfolding of SOD1 and assess pathways that could not be studied with other bioanalytical techniques. By applying multiple solution conditions to systematically denature the holo-dimer, first elongation was observed, followed by dissociation into holo-, single-metal, and apo-monomeric SOD1 units. Loss of the metal cofactors was found to further destabilize the protein monomers, ultimately resulting in formation of extremely extended apo-monomers. The results obtained from IMS-MS in these study are extremely important as perturbations observed from SOD1 in disease states may not be readily measured by NMR or X-ray crystallography if present in low concentrations or convoluted by the potential presence of multiple conformers. Thus, IMS-MS may have particular utility for examining samples from patients with certain disease states, including ALS. In addition, combining the ability to directly probe SOD under native conditions directly from tissue (Roberts et al., 2007; Rhoads et al., 2011) allows for the measurement of structural properties of the enzyme without lengthy purification process that may alter the state of the enzyme. Combined with collision induced unfolding this opens up the possibility of directly probing the stability and structural properties of SOD1 from human familial ALS tissue and mouse models. Furthermore, IMS-MS has the potential to explore the biophysical properties of purified proteins or recombinant systems and rapidly compare mutations, drug binding or other parameters thought to disturb structure of a protein and loosely relate this to NMR and crystallographic data, and thus provides complementary information to these more conventional solution-based experiments. As there are currently over 150 known mutations of human SOD1, and it is thought that loss of the metals from the holo-dimeric protein result in the apo-protein and contribute to the formation of plaques observed in ALS, the ability to assess the unfolding characteristics with IMS-MS may facilitate an understanding of how they alter protein dynamics.

## Data Availability

Raw data is available through MassIVE (https://massive.ucsd.edu/MSV000086204).
