# Supplementary material for: Utilizing Ion Mobility-Mass Spectrometry to Investigate the Unfolding Pathway of Cu/Zn Superoxide Dismutase
Source: Front Chem. 2021 Feb 9;9:614595. doi: 10.3389/fchem.2021.614595 (PMC7900566; doi:10.3389/fchem.2021.614595)
Supplement: Supplementary file 2 [file table2.docx]

Supplementary Material

**Utilizing Ion Mobility-Mass Spectrometry to Investigate the Unfolding Pathway of Cu/Zn Superoxide Dismutase**

**Karen E. Butler ^1^, Yoshihiko Takanami^2^, Adam Rainczuk^3^, Erin S. Baker^1*^, Blaine R. Roberts^4,5*^**

^1^North Carolina State University, Department of Chemistry, Raleigh, NC, United States

^2^Bruker Japan, K.K. 3-9, Moriya-cho, Kanagawa-ku, Yokohama City, Kanagawa, Japan

^3^Bruker Pty Ltd, Preston, Victoria, Australia

^4^Department of Biochemistry, Emory University School of Medicine, Atlanta, GA, United States

^5^Department of Neuroscience, Emory University School of Medicine, Atlanta, GA, United States

**Figures S1-S6** summarize experimental parameters for the Bruker TIMS-TOF MS instrument.

**Tables S1-S3** summarize experimental parameters for the Agilent 6560 IM-QTOF MS platform.

**Figures S7-S9** illustrate supplemental data from both the Agilent 6560 IMS-QTOF MS (**Figure S7**) and Bruker TIMS-TOF MS **(Figures S8-S9).**


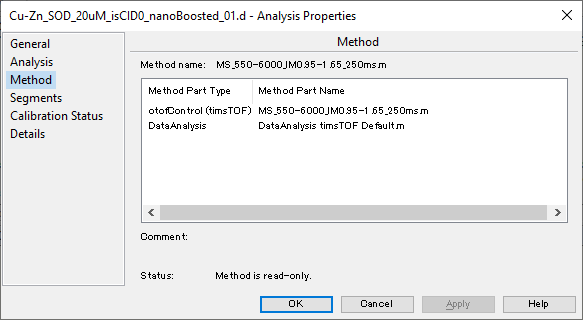


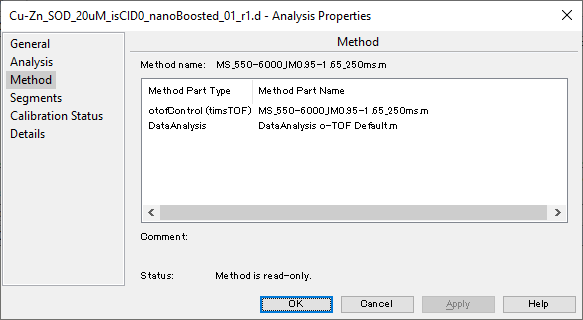


**Supplementary Figure S1.** General method screen for the Bruker TIMS-TOF acquisition showing the method name.


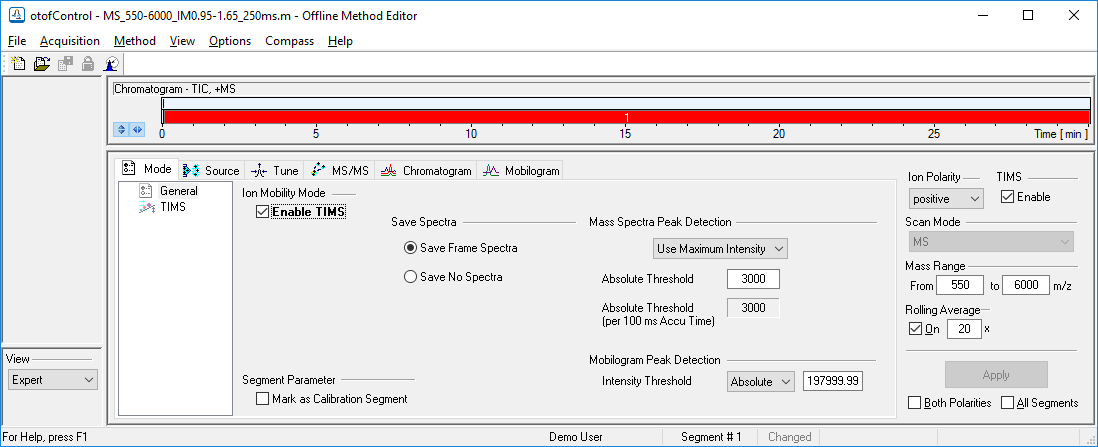


**Supplementary Figure S2.** General mode selection for the Bruker TIMS-TOF method.


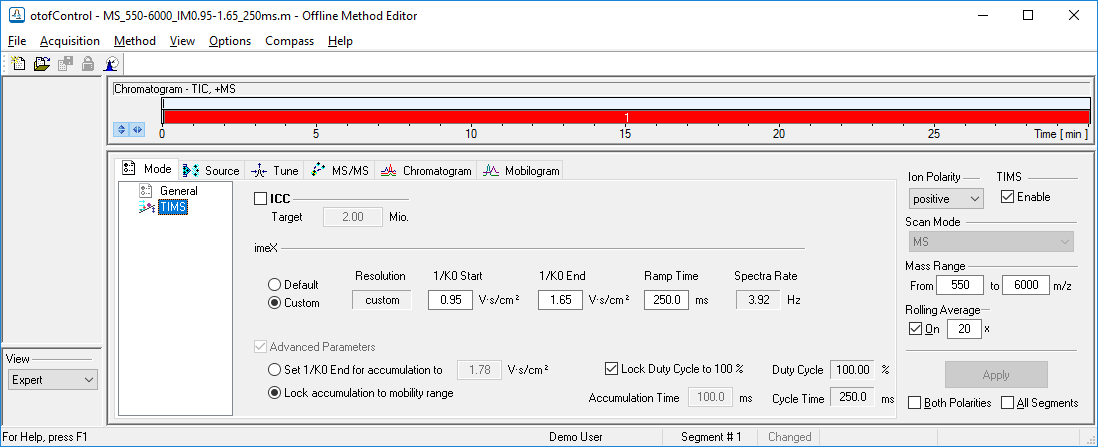


**Supplementary Figure S3.** Parameters for TIMS acquisition within the “Mode” tab of the Bruker TIMS-TOF method editor.


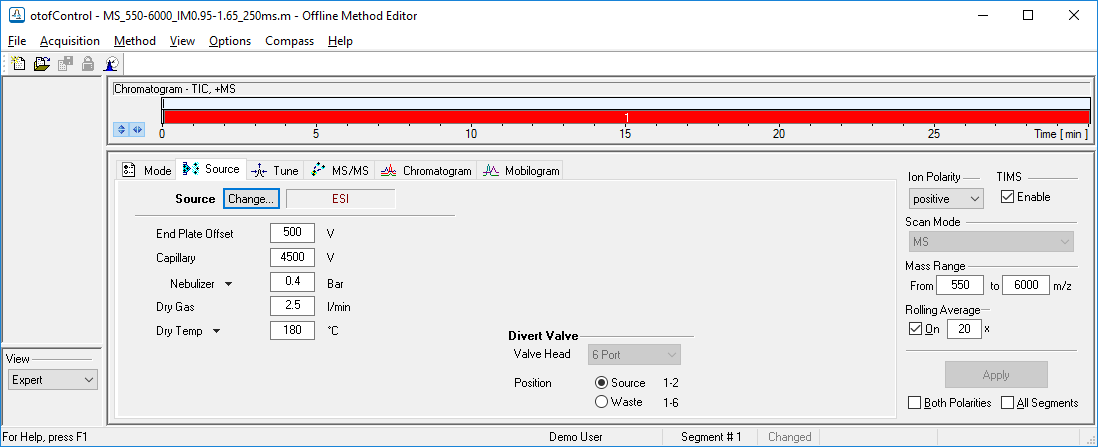


**Supplementary Figure S4.** Source parameters for Bruker TIMS-TOF IMS-MS data acquisition.


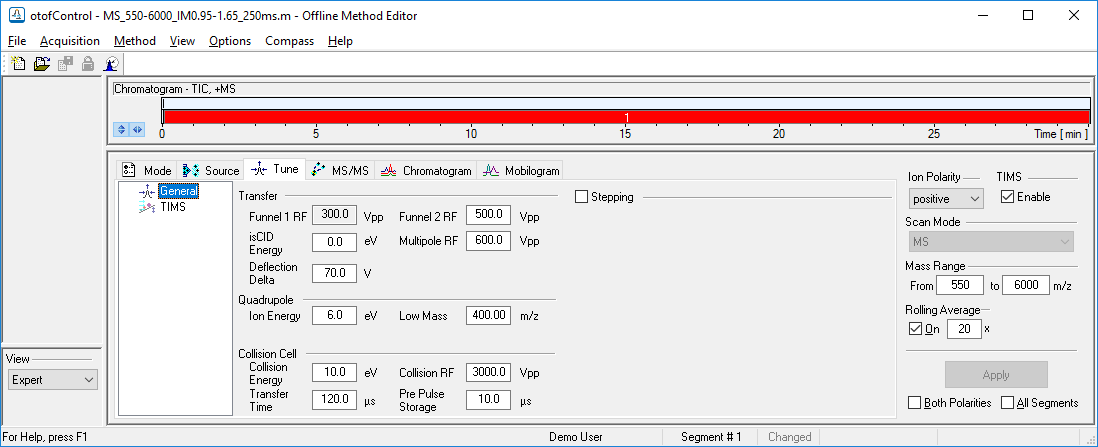


**Supplementary Figure S5.** General tune parameters used for acquisition of TIMS-TOF data using the Bruker TIMS-TOF.


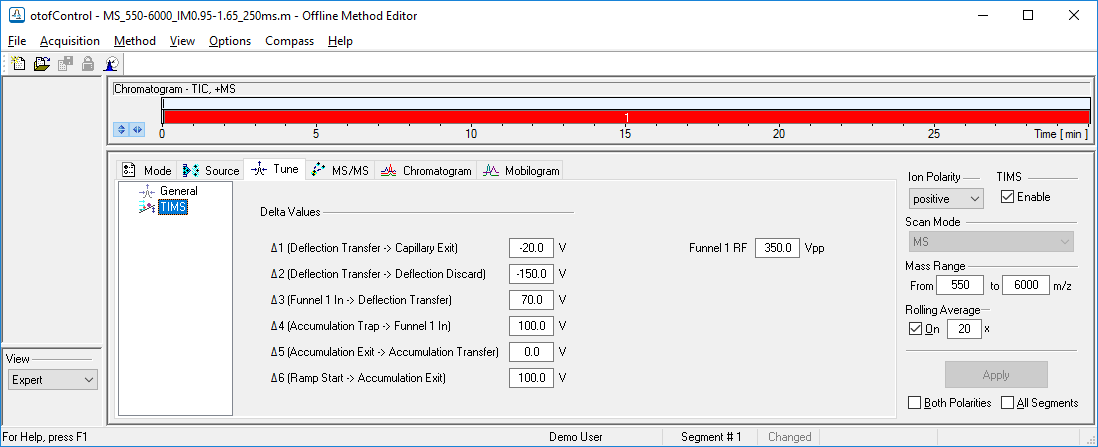


**Supplementary Figure S6.** TIMS-specific tune parameters used for acquisition of TIMS-TOF data using the Bruker TIMS-TOF.

**Supplementary Table S1.** Source conditions used for acquisition of DTIMS-MS data using the Agilent 6560.

| **Source Conditions** | |
| --- | --- |
| Gas Temperature | 300 ⁰C |
| Drying Gas | 5 L/min |
| Capillary Voltage | 1500 V |
| Flow rate (syringe pump) | 300 nL/min |

**Supplementary Table S2.** IM drift tube settings used for acquisition of DTIMS-MS data using the Agilent 6560.

| **IM Drift Tube Settings** | |
| --- | --- |
| DT Entrance Voltage | 1574 V |
| DT Exit Voltage | 224 V |

**Supplementary Table S3.** Data acquisition parameters used for collection of DTIMS-MS data using the Agilent 6560.

| **Acquisition Parameters** | |
| --- | --- |
| Mass Range | 100-20,000 *m/z* |
| IM Trap Fill Time | 40 ms |
| IM Trap Release Time | 200 µs |
| Max Drift Time | 100 ms |
| Multiplexing Mode | Disabled |


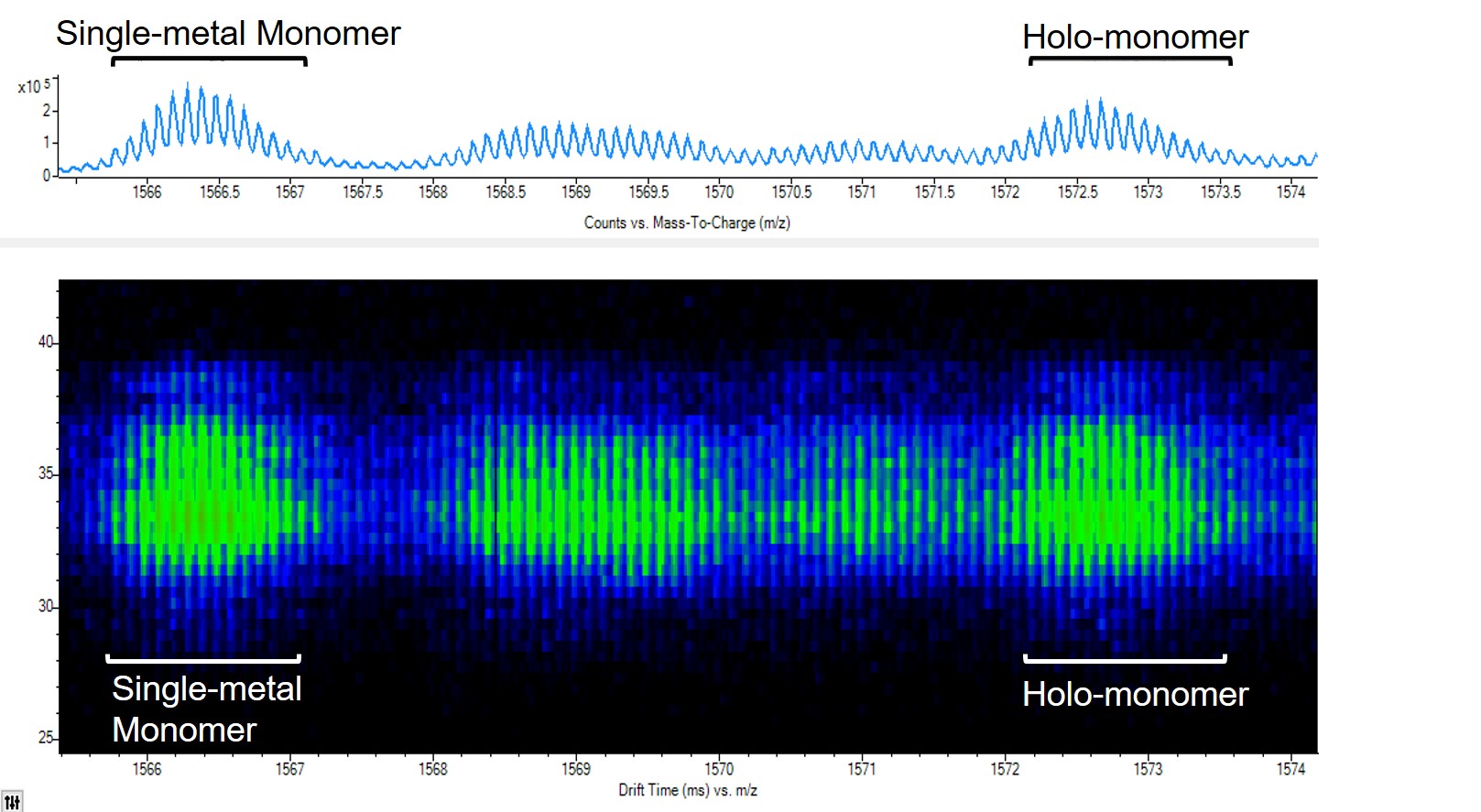


| **Bovine SOD (P00442)** | | |
| --- | --- | --- |
|  | **Chemical Formula (10+ Charge State)** | **Exact Mass** |
| **Holo Cu, Zn SOD** | **CuZnC_672_H_1081_N_198_O_221_S_4_** | **15715.6903** |
| **Cu-only SOD** | **CuC_672_H_1083_N_198_O_221_S_4_** | **15653.7768** |
| **Zn-only SOD** | **ZnC_672_H_1083_N_198_O_221_S_4_** | **15654.7763** |
| **Apo SOD** | **C_672_H_1085_N_198_O_221_S_4_** | **15592.8628** |

**Supplementary Figure S7.** Zoomed in nested IMS-MS spectrum from the Agilent DTIMS-TOF instrument showing the holo-monomer and single-metal monomer in the 10+ charge state, observed in the 30% acetonitrile (ACN), 100 µM formic acid (FA) solution condition. A list of the chemical formulas and exact masses for the SOD monomer proteoforms in their 10+ charge state is shown. The calculations of chemical formula were done with N-terminal methionine removed and N-terminus acetylated, one disulfide bond and protons added to reflect the 10+ charge state with respect to Cu^2+^ and Zn^2+^.


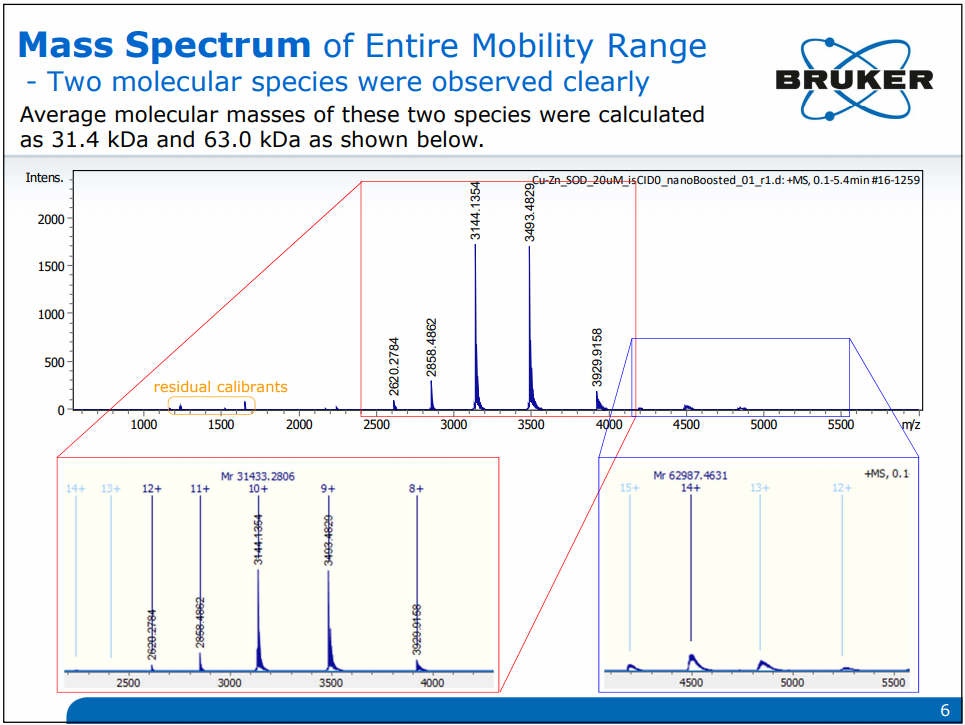


**Supplementary Figure S8.** Data obtained from the Bruker TIMS-TOF platform for the native conditions of SOD1. The average mass of the observed SOD1 holo-dimer was calculated to be 31.4 kDa.


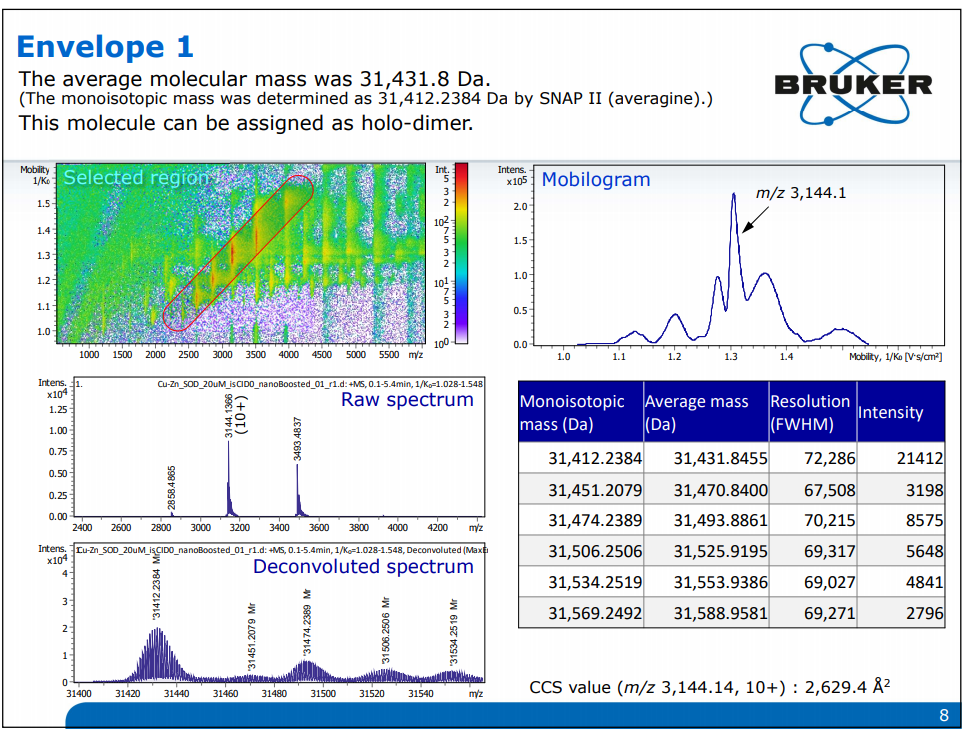


**Supplementary Figure S9.** Zoomed in mass spectrum from the Bruker TIMS-TOF instrument showing the holo-dimer in the 10+ charge state, along with the deconvoluted spectrum.
